# Type 1 Cytotoxic T Cells Increase in Placenta after Intrauterine Inflammation

**DOI:** 10.3389/fimmu.2021.718563

**Published:** 2021-09-08

**Authors:** Jin Liu, Yang Liu, Snigdha Panda, Anguo Liu, Jun Lei, Irina Burd

**Affiliations:** Integrated Research Center for Fetal Medicine, Department of Gynecology and Obstetrics, Johns Hopkins University School of Medicine, Baltimore, MD, United States

**Keywords:** intrauterine infection/inflammation, placenta, CD8+ T cell subsets, interferon gamma, Tc1/Tc2

## Abstract

CD8+ T cells recognize non-self antigen by MHC class I molecules and kill the target cells by the release of proinflammatory cytokines such as interferon gamma (IFN-γ) and tumor necrosis factor alpha (TNF-α). Our group previously reported an increase of CD8+ T‐cell trafficking in the placenta with exposure to Lipopolysaccharides (LPS). CD8+ cytotoxic T cells have been classified into distinct subsets based upon cytokine production: Tc1 cells produce IFN-γ, Tc2 cells produce interleukin 4 (IL-4). Accordingly, the purpose of this research is to analyze the subsets of placenta CD8+ T cells. We hypothesized that LPS injection would induce a change of properties of CD8+ T cell and Tc1/Tc2 ratio. We investigated the subsets of CD8+ T cell infiltration to placenta and their specific function in response to LPS-induced inflammation in a mouse model. At embryonic (E) day 17, pregnant CD-1 dams received an intrauterine injection of 25 µg LPS in100 μl PBS or 100 μl of PBS only. Flow cytometry was used to quantify CD8+ T cells, evaluate the phenotype and subtypes, and detect markers of Tc1 and Tc2 cells in placenta, at 6 hours and 24 hours post injection (hpi). Intracellular staining and flow cytometry were performed to characterize cytokines produced by CD8+ T cells. Standard statistical analysis were employed. After 6 and 24 hours of LPS injection, total CD8 T cells increased (P<0.05). Tc1 cells expanded (P<0.05) in LPS-treated dams compared with the PBS group. The Tc1/Tc2 ratio was significantly higher in the LPS group than the PBS group (P<0.05). The expression of TNF-α and IFN-*γ* were increased in LPS group both at 6hpi and 24 hpi (P<0.05). We identified functional placental CD8+ T cell subtypes and found a significant increase ratio of Tc1/Tc2. Following IUI, CD8+ T cells induced inflammatory response in the placenta primarily *via* the production of Type 1 cytokines such as IFN-γ and TNF-α. We have provided evidence of a Tc1-bias response and cytokines in the mouse model of IUI.

## Introduction

Villitis of unknown etiology (VUE) is a type of intrauterine inflammation and destructive inflammatory lesion resulting in placental destruction and dysfunction, which is characterized by the infiltration of maternal T cells-especially cytotoxic CD8+ T cells-into the placenta, specifically into the chorionic villi (fetal tissue) (1). VUE is a crucial cause of preeclampsia, intrauterine growth restriction, small-for-gestational-age fetuses, fetal/neonatal death, spontaneous preterm delivery and recurrent reproductive loss, and neonatal neurocognitive impairment (2–5). VUE is also believed to destruct large fetal vessels in the placenta leading to obliterative fetal vasculopathy, which is a high-risk factor for neonatal encephalopathy and cerebral palsy (6, 7).

Considering its high recurrence risk (10-37% (8, 9),) and poor clinical outcomes, it is vital to establish a diagnosis and effective management to counsel affected women in their subsequent pregnancy. Thus, greater understandings of the pathophysiology and pathogenesis of VUE are essential to understand its contribution to the adverse outcomes and to establish effective treatment guidelines.

In VUE, the causes of chronic inflammatory lesions are unknown, and infectious agents cannot be identified (2). Accumulating studies suggest that an immune process caused by maternal anti-fetal rejection plays a role in the pathogenesis of this condition, in which cytotoxic CD8+ T cells are the main effector cells (5, 10).

CD8+ T cells recognize non-self antigens by MHC class I molecules and kill the target cells by releasing proinflammatory cytokines such as IFN-*γ* and TNF-*α* (11). Based on its pattern of cytokines production, activated CD8+ T cells can be classified into Tc1 and Tc2 subsets, which are parallel with Th1 and Th2 subsets. Type 1 CD8+ T cells (Tc1) secrete IFN-γ and TNF-α, whereas type 2 CD8+ T cells (Tc2) secrete IL-4, IL-5 and IL-13 (12). One human study reported that the immune response in the VUE lesions is directed toward the Th1-type response (13). Subset of CD8+ T cells and type of immune and inflammatory response that mainly contribute to this process remains unknown.

Our group previously utilized a mouse model of lipopolysaccharide (LPS)-induced intrauterine inflammation (IUI) to study maternal T cells infiltration to the placenta and the effect of placental T cells on placental dysfunction, inflammation and subsequent offspring’s sequela (14, 15). The aim of this study is to investigate the specific functional properties of different CD8+ T cell subsets infiltrating in the placenta by using a mouse model of LPS-induced IUI.

## Materials and Methods

### Animal Model and Experimental Groups

All animal care and treatment procedures were approved by the Animal Care and Use Committee of the Johns Hopkins University. All methods were performed in accordance with the relevant guidelines and regulations of the Johns Hopkins University. Timed-pregnant CD-1 mice were purchased from Charles River Laboratories (Wilmington, MA, USA).

A well-established model of IUI was utilized in this study (15–18). On embryonic day (E) 17 of gestation (full-term gestation is E19-20), CD-1 mice were randomly assigned to LPS or phosphate-buffered saline (PBS) groups. After placing the mouse under isoflurane anesthesia, a laparotomy was performed in the lower abdomen. The lower right uterine horn was identified and either LPS (from E. coli O55: B5; Sigma-Aldrich, St Louis, MO, USA) at a dose of 25µg in 100 µl PBS or 100 µL PBS (control) was injected. Then routine laparotomy closure was performed, and the mice were allowed to recover. Mice were randomly selected to be euthanized by CO_2_ exposure followed by cardiac exsanguination 6 hours and 24 hours after the surgery. At the time of euthanasia, the total number of viable and dead fetuses was quantified for each dam.

### Fetal Viability

Live and dead fetuses of right side in each dam were measured. Dead fetus was determined by its movability alterations and other abnormalities including pallor body and foci of necrotic tissue in the placenta (19).

### Immunofluorescence Imaging

Tissues were fixed in 4% paraformaldehyde for 24h at 4 °C. Tissues were then washed with PBS extensively and immersed in 30% sucrose until saturation, followed by cryosection at 10 μm thickness. Antigens were retrieved by boiling in PBS buffer () for 20 min. After antigen retrieval, tissues were blocked in 10% goat serum and permeabilized with 0.5% Triton-X-100. Tissues were incubated with primary antibodies overnight at 4 °C. The following primary antibody was used: CD8 alpha recombinant rabbit monoclonal antibody (1:100, MA5-29682, Invitrogen) to identify CD8+ T cells. The next day tissues were washed with PBS, followed by secondary antibody incubation for 1 h at room temperature. Secondary goat antibody was used: anti-rabbit IgG (1:500, A10042, Invitrogen) Alexa Fluor 568. DAPI (4′,6-diamidino-2-phenylindole, 10236276001, Roche, Indianapolis, IN) was used to counter stain nuclei at a concentration of 1:5,000 Slides were mounted with Fluoro-mount G (SouthernBiotech, Birmingham, AL) and viewed using a Zeiss Axioplan 2 microscope (Jena, Germany). Images were taken using a Zeiss AxioCam MRM.

### Flow Cytometry

Three placentae were harvested for flow cytometry, from the first three gestational sacs in the right uterine horn at 6 and 24 hours after surgery. Single cells were prepared from 3 placentae per dam, using manual and enzymatic (collagenase D, Roche, Indianapolis, IN, USA) digestion, followed by passing through a 70 µm nylon mesh cell strainer2-4. Red blood cell lysis (ACK lysing buffer, ThermoFisher Scientific, MA, USA) and total cell counts were performed prior to staining. Isolated cells were stained with various surface markers, at 1:100 concentration, in fluorescence activated cell sorting (FACS) buffer with 1Mm EDTA for 30 min at 4°C protecting from light. For intracellular staining, cells were first fixed with 1X FOXP3 Fix/Perm (BioLegend, San Diego, CA, USA) and then stained with intracellular markers for 45 min at a concentration of 1:50 at 4°C in the dark. OneComp eBeads (eBioscience, San Diego, CA, USA) were used for single-color compensation controls.

Antibodies and corresponding isotypes are listed in **Table 1**. In this study, CD3 was applied as a pan T cell marker and CD8α as a CD8+ T cell marker (20). CD44 and CD62L were utilized to gate effector sub-population (21). Tim-3 was used to identify CD8+ T cell exhaustion (22). IFN-*γ* and IL-4 were used to define Tc1 and Tc2. Data was acquired from an Attune NxT Acoustic Focusing Cytometer (ThermoFisher Scientific Invitrogen) and analyzed with FlowJo 10.1 (FlowJo LLC, Ashland, OR, USA). Debris and doublets were excluded by sequential gating on side scatter height *versus* side scatter area to delaminate singlets. Seven-color flow cytometry was used to identify CD8+ T cell sub-populations.

**Table 1:** Antibodies List.

Antibodies	Clone	Isotype	Company	Catalog No.
PE Rat Anti-mouse CD45	30-F11	Rat/IgG2b, kappa	BD Pharmingen	553081
eFluor 450 Anti-mouse CD3	17A2	Rat/IgG2b, kappa	Invitrogen	48-0032-82
Super Bright 600 Anti-mouse CD8a	53-6.7	Rat/IgG2a, kappa	Invitrogen	63-0081-82
eFluor 450 Anti-mouse CD8a	53-6.7	Rat/IgG2a, kappa	Invitrogen	48-0081-82
Mouse CD44 Alexa Fluor 405	IM7.8.1	Rat/IgG2b	Invitrogen	RM5726
Anti-mouse CD62L Super Bright 600	MEL-14	Rat/IgG2a, kappa	Invitrogen	63-0621-82
Anti-mouse CD366 (TIM3) PE-Cyanine7	RMT3-23	Rat/IgG2a, kappa	Invitrogen	25-5870-82
Anti-mouse IFN gamma PE-Cyanine7	XMG1.2	Rat/IgG1, kappa	Invitrogen	25-7311-82
Anti-mouse TNF alpha PE-Cyanine7	MP6-XT22	Rat/IgG1, kappa	Invitrogen	25-7321-82
Anti-mouse IL-4 Alexa Fluor 488	11B11	Rat/IgG1, kappa	Invitrogen	53-7041-82
Anti-mouse IL-13 Alexa Fluor 488	eBio13A	Rat/IgG1, kappa	Invitrogen	53-7133-82
CD8 alpha recombinant rabbit monoclonal antibody	------	Rabbit / IgG	Invitrogen	MA5-29682

### Statistical Analysis

Fetal viability data were assessed with a Chi-square test. The normality of continuous variables was examined by QQ plot, D’Agostino-Pearson, Anderson-Darling, Shapiro-Wilk and Kolmogorov-Smirnov. Outliers were identified using Grubb’s test. Two-way analysis of variance (ANOVA) with Sidak’s multiple comparisons test was used for parametric data analysis. Correlations were analyzed using Pearson correlation test. All data were analyzed with GraphPad Prism 7 software (Graphpad Software, La186 Jolla, CA, USA). Data are expressed as mean ± SD. Results were deemed statistically significant when p<0.05.

## Results

### LPS Intrauterine Administration During Pregnancy Induced Lymphocytic Infiltration in the Placenta and Reduced Offspring Survival

**Figure 1:**
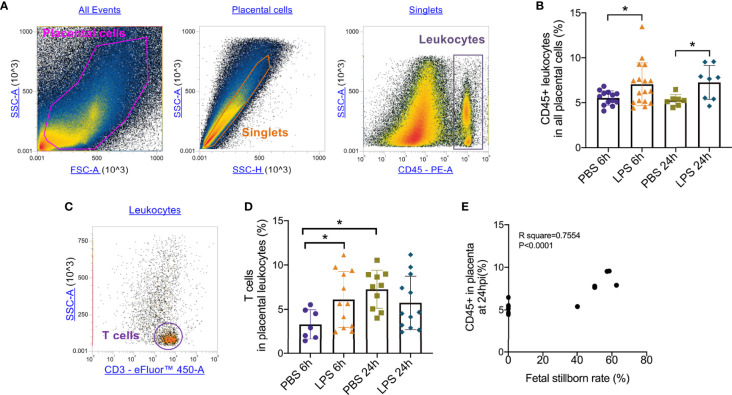
Placental leukocytes infiltration and correlation with fetal demise. **(A)** All captured placental cells were distinguished based on properties of side scatter area (SSC-A) *versus* forward scatter area (FSC-A) generally, by using polygon/rectangular/oval gates. Debris and doublets were excluded by gating on side scatter height (SSC-H) *versus* SSC-A in order to further delaminate singlets. Placental leukocytes were gated sequentially on CD45+ *versus* SSC-A properties. **(B)** Placentae were collected at 6 and 24hpi. 6 and 24 hours after LPS exposure, CD45+ leukocytes infiltrated in the placenta were increased (*p < 0.05; at 6hpi, LPS: 7.06 ± 2.38%, n = 17; Control: 5.52 ± 0.79%, n = 13. at 24hpi, LPS: 7.24 ± 1.88%, n = 8; Control: 5.24 ± 0.64%, n = 7). **(C)** The CD3+ T cells were then further identified by CD3+ and SSC-A properties. **(D)** At 6hpi, CD45+CD3+ T cells in LPS group were increased compared to control (*p < 0.05; LPS: 6.11 ± 3.14%, n = 12; Control: 4.84 ± 2.83%, n = 10); at 24hpi, no changes between LPS and PBS group were found (p > 0.05; LPS: 5.734 ± 3.02%, n = 2; Control: 7.26 ± 2.15%, n = 10). In PBS group, T cells infiltrated increasingly at 24hpi compared to 6hpi. Data were reported as Mean ± SD. **(E)** We then analyzed correlations. Offspring viability was significantly correlated to CD45+ in the placenta at 24hpi (p < 0.0001; R^2^ = 0.7554). Two-way ANOVA and Pearson’s r test were used.

To test the role of LPS intrauterine treatment in lymphocytic infiltration in the placenta, flow cytometry was performed. We first analyzed leukocytes in the placenta. Gate strategy was shown in **Figure 1A**. 6 and 24 hours after LPS exposure, CD45+ leukocytes infiltrated in the placenta were increased (p<0.05, **Figure 1B**). Then we gated T cell to analyze T cell frequency in the placenta **(** **Figure 1C** **)**. At 6hpi, CD45+CD3+ T cells were increased (p<0.05) but no changes were found at 24hpi compared to the control group (**Figure 1D**). Interestingly, placental T cells in control group increased at 24hpi (E18.5) compared to that at 6hpi (E17.5), while in LPS group, there were no changes (**Figure 1D**). The data illustrated that T cells may play an essential role in the placenta during parturition following LPS intrauterine administration. In addition, we investigated the correlation of the fetal viability and leukocytic/lymphocytic infiltration into the placenta at 6hpi and 24hpi (**Table 2**). There was a significant negative correlation between placental CD45+ leukocytes and fetal viability 24 hours after LPS administration (**Figure 1E**). To examine the effectiveness of LPS intrauterine treatment in fetal viability, we then measured live and dead fetuses of right side in each dam. Offspring viability was significantly decreased in LPS-administrated dams compared to that in PBS-treated dams at 6hpi or 24hpi (p<0.001, **Figure S1**).

**Table 2:** Pearson R test for fetus viability and leukocytic/lymphocytic infiltration into the placenta.

	6hpi			24hpi	
	Stillborn rate				
	Leukocytes		T cells	Leukocytes	T cells
r	-0.2133	0.1198		0.8691	-0.3853
R squared	0.0455	0.01435		0.7554	0.1484
P (two-tailed)	0.4453	0.6707		<0.0001	0.1561
P value summary	ns	ns		****	ns
Significant? (alpha = 0.05)	No	No		Yes	No
Number of XY Pairs	15	15		15	15

****: p < 0.0001. ns, not significant (p > 0.05).

### Placental CD8+ T Cells and Subsets Were Altered Following Intrauterine LPS Administration

**Figure 2:**
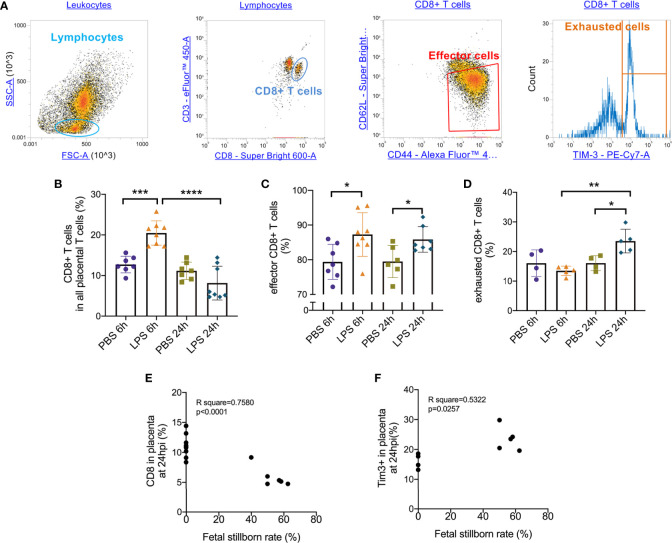
Effector/exhausted status of placental CD8+ T cells. Placental cells were stained with CD3, CD8, CD44, CD62L and Tim-3. Sequential gating was utilized to identify specific CD8 T cell effector.exhausted properties. **(A)** The placental lymphocytes were determined by forward and side scatter sequentially on placental leukocytes. CD3+ CD8+ population was defined as CD8+ T cell. CD44^high^ CD62L^low^ population was defined as effectors. Tim-3+ cells were defined as exhausted cells. **(B)** There was an increase of CD3+CD8+ T cell frequency at 6hpi but no significance at 24hpi. In LPS group, CD8+ T cells increased at 24hpi compared to 6hpi (***p < 0.001, ****p < 0.0001; at 6hpi, LPS: 20.47 ± 2.99%, n = 8; Control: 12.71 ± 2.04%, n = 7. at 24hpi, LPS: 8.151 ± 4.16%, n = 8; Control: 11.15 ± 2.15%, n = 7). **(C)** At 6hpi and 24hpi, effector CD8+ T cell frequency increased (*p < 0.05; at 6hpi, LPS: 87.28 ± 6.29%, n = 8; Control: 79.39 ± 5.06%, n = 7. at 24hpi, LPS: 85.85 ± 3.70%, n = 7; Control: 79.49 ± 4.64%, n = 6). **(D)** No significant changes were found in exhausted CD8+ T cells at 6hpi (p > 0.05; LPS: 13.48 ± 1.57%, n = 5; Control: 16.05 ± 4.48%, n = 4) but there was an increase at 24hpi (*p < 0.05; LPS: 23.52 ± 4.01%, n = 5; Control: 16.06 ± 2.51%, n = 4). In LPS group, exhausted CD8+ T cells increased at 24hpi compared to 6hpi. **(E, F)** There were weak correlations between CD8+ T cell and its exhaustion and fetal stillborn rate at 24hpi (p < 0.05, R^2^ = 0.5322). Data were reported as Mean ± SD. Two-way ANOVA and Pearson’s r test were used. **p < 0.01.

Following the observation that placental T cell infiltration was changed by LPS, we performed flow cytometry to further analyze CD8+ T cell and its effector and exhausted status. Gating strategy was shown in **Figure 2A**. 6 hours after intrauterine LPS exposure, there was an increase of CD3+CD8+ T cell frequency (p<0.05, **Figure 2B**) and effector CD8+ T cell frequency (p<0.05, **Figure 2C**) but no significant change for exhausted CD8+ T cells (**Figure 2D**). Following LPS exposure, placental effector and exhausted CD8+ T cell frequency increased at 24hpi. (p<0.05, **Figures 2C, D**). In LPS group, CD8+T cells decreased from 6hpi (E17.5) to 24hpi (E18.5) while exhausted CD8+ T cells increased from E17.5 to E18.5 compared to that in control group, which could be speculated that more exhaustion of CD8+T cells in placenta happened during parturition with LPS intrauterine exposure (**Figures 2B, D**). Then we investigated the correlation of the fetal viability and CD8+ T cell and its status in the placenta at 6hpi and 24hpi (**Table 3**). There were correlations between CD8+ T cells exhaustion and fetal stillborn rate at 24hpi (**Figures 2E, F**). These results demonstrated that LPS may induce the effector function and trigger exhausted status of CD8+ T cell, and exhaustion was proved to have negative impact on fetal viability.

**Table 3:** Pearson R test for fetus viability and CD8+ T cell and its status in the placenta.

	6hpi				24hpi		
	Stillborn rate *vs*.						
	CD8 T cell	Effector cells	Exhausted cells		CD8 T cell	Effector cells	Exhausted cells
r	0.3635	0.4984	-0.3202		-0.8706	0.07154	0.7295
R squared	0.1321	0.2484	0.1025		0.758	0.005117	0.5322
P (two-tailed)	0.183	0.0586	0.4009		<0.0001	0.8	0.0257
P value summary	ns	ns	ns		****	ns	*
Significant? (alpha = 0.05)	No	No	No		Yes	No	Yes
Number of XY Pairs	15	15	9		15	15	9

*p < 0.05, ****p < 0.0001.

ns, not significant (p > 0.05).

**Figure 3:**
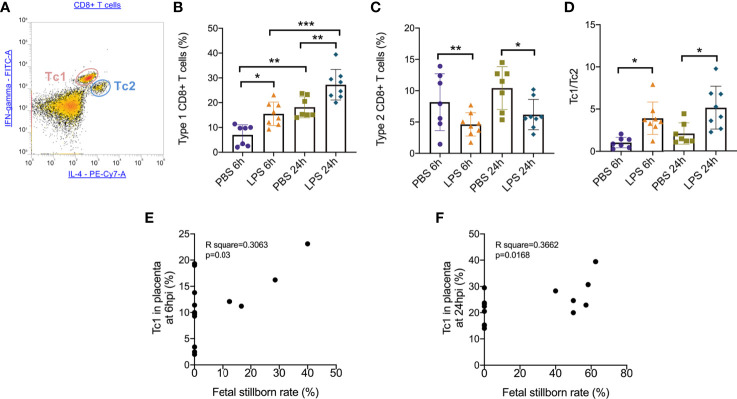
Subsets of effector CD8+ T cells trafficking in the placenta following LPS infusion. **(A)** The relative expression of IFN-*γ* and IL-4 to identify Tc1 (IFN-*γ* ^high^ IL-4^low^) and Tc2 (IFN-*γ* ^low^ IL-4^high^) were then determined on CD8+ T cell populations. **(B)**At both 6hpi and 24hpi, the frequency of Type 1 CD8+ T cells (Tc1) increased. Tc1 was also increased by time regardless LPS administration. (*p < 0.05, **p < 0.01, ***p < 0.001; at 6hpi, LPS: 15.50 ± 4.73%, n = 8; Control: 6.96 ± 4.11%, n = 7. At 24hpi, LPS: 27.23 ± 6.18%, n = 8; Control: 18.14 ± 4.22%, n = 7). **(C)**Type 2 CD8+ T cells (Tc2) decreased at 6hpi (*p < 0.05; Student’s t test; LPS: 4.62 ± 1.87%, n = 8; Control: 8.17 ± 4.54%, n = 7) and at 24hpi (*p < 0.05; Student’s t test; LPS: 6.18 ± 2.42%, n = 8; Control: 10.41 ± 3.42%, n = 7). **(D)** The ratio of Tc1 to Tc2 increased at both 6hpi (**p < 0.01; Student’s t test; LPS: 3.91 ± 1.92, n = 8; Control: 1.03 ± 0.63; n = 7) and 24hpi (*p < 0.05; LPS: 5.14 ± 2.54, n = 8; Control: 2.07 ± 1.27; n = 7). **(E, F)** There were weak correlations between Tc1 and fetal stillborn rate at 6hpi and 24hpi (p < 0.05, R^2 ^= 0.3662). Data were reported as Mean ± SD. Two-way ANOVA and Pearson’s r test were used.

To further determine the specific mechanism of effector CD8+ T cells that function in the placenta, we gated (**Figure 3A**) and analyzed the subtype of effector CD8+ T cells. At both 6hpi and 24hpi, the frequency of Type 1 CD8+ T cells (Tc1) increased (p<0.01, **Figure 3B**) while Type 2 CD8+ T cells (Tc2) decreased (p<0.05, **Figure 3C**), and the ratio of Tc1 to Tc2 increased (p<0.01, **Figure 3D**). Furthermore, from E17.5 to E18.5, Tc1 increased with or without LPS exposure (p<0.05), while no changes were observed for Tc2 or Tc1/Tc2 (**Figures 3B–D**). These results demonstrated that the Type 1 CD8+ T cells are mainly responsible for the inflammatory cytotoxic responses following LPS exposure. Furthermore, we examined the correlations between fetal stillborn rate and Tc1, Tc2 (**Table 4**). The percentage of placental Tc1 had negative impact on fetal viability at 6hpi and 24hpi (**Figures 3E, F**).

**Table 4:** Pearson R test for fetus viability and Tc1, Tc2 in the placenta.

	6hpi			24hpi		
	Stillborn rate *vs*.					
	Tc1	Tc2	Tc1/Tc2	Tc1	Tc2	Tc1/Tc2
r	0.5534	-0.3025	0.4115	0.6051	-0.2896	0.2707
R squared	0.3063	0.09148	0.1693	0.3662	0.08388	0.07325
P (two-tailed)	0.0323	0.2732	0.1276	0.0168	0.2951	0.3292
P value summary	*	ns	ns	*	ns	ns
Significant? (alpha = 0.05)	Yes	No	No	Yes	No	No
Number of XY Pairs	15	15	15	15	15	15

*p < 0.05.

ns, not significant (p > 0.05).

**Figure 4:**
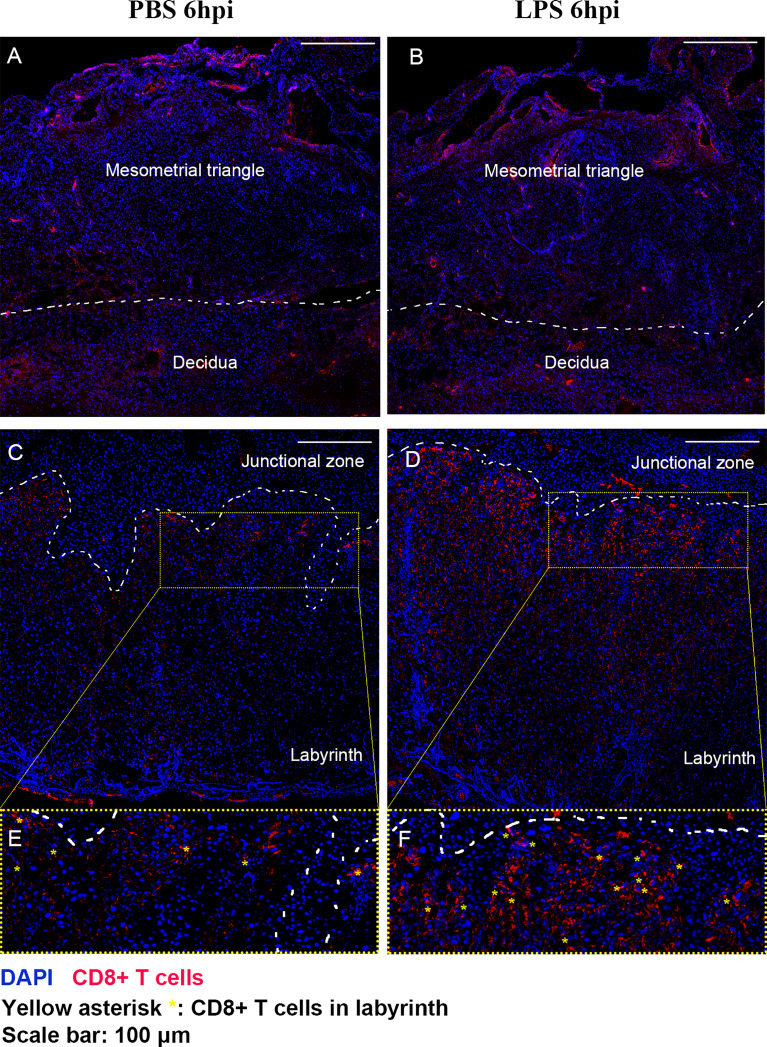
The distribution of CD8+ T cells in the placenta following LPS exposure. IF staining was performed 6 hours after surgery to investigate the distribution of CD8+ T cells in the placenta following LPS exposure. **(A, B)** Regardless of whether LPS or PBS was injected, CD8+ T cells appeared to infiltrate in mesometrial triangle and decidua. **(C–F)** CD8+ T cells were localized primarily in labyrinth tissue near junctional zone, following LPS exposure.

We further performed IF staining 6 hours after surgery to investigate the distribution of CD8+ T cells in the placenta following LPS exposure (**Figure 4**). Regardless of whether LPS or PBS was injected, CD8+ T cells appeared to infiltrate in mesometrial triangle and decidua (**Figures 4A, B**). CD8+ T cells were localized primarily in labyrinth tissue near junctional zone, following LPS exposure (**Figures 4C–F**).

### Cytokines Produced by Tc1 and Tc2 Were Altered Following IUI Exposure

**Figure 5:**
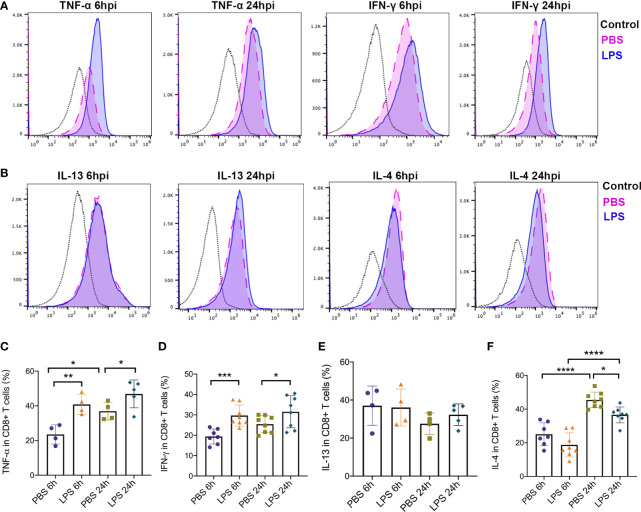
Type 1 and Type2 cytokines produced by effector CD8+ T cells. Intracellular staining was performed to detect expression of cytokines produced by CD8+ T cells. **(A)** MFI and positive-stained cells in CD8+ T cells of TNF-α and IFN-*γ* were calculated by Flowjo. **(B)** MFI and positive-stained cells in CD8+ T cells of IL-4 and IL-13 were calculated by Flowjo. **(C)** At both 6hpi and 24hpi, the expression of TNF-α in effector CD8+ T cells was elevated. In PBS group, TNF-α increased at 24hpi compared to 6hpi. (**p < 0.01, *p < 0.05; at 6hpi, LPS: 40.83 ± 5.70%, n = 4; Control: 23.53 ± 5.53%, n = 4. At 24hpi, LPS: 46.88 ± 8.04%, n = 5; Control: 36.95 ± 5.09%, n = 4) **(D)** IFN-γ in placental CD8+ T cells increased (***p < 0.001, *p < 0.05; at 6hpi, LPS: 29.68 ± 5.16%, n = 8; Control: 19.37 ± 3.67%, n = 7. At 24hpi, LPS: 31.51 ± 7.88%, n = 8; Control: 25.42 ± 4.15%, n = 8). **(E)** For IL-13, no significant changes were found at both 6hpi and 24hpi (p > 0.05; at 6hpi, LPS: 36.08 ± 9.73%, n = 4; Control: 37.05 ± 10.34%, n = 4. At 24hpi, LPS: 32.30 ± 5.71%, n = 5; Control: 27.55 ± 5.64%, n = 4). **(F)** There was a significant decrease of IL-4 at 24hpi (*p < 0.05, ****p < 0.0001; LPS: 36.58 ± 4.70%, n = 8; Control: 45.48 ± 4.66%, n = 8) but no statistical significance at 6hpi (p > 0.05; LPS: 18.72 ± 7.17%, n = 8; Control: 25.02 ± 6.85%, n = 7). IL-4 was increased by time regardless LPS administration. Data were reported as Mean ± SD. Two-way ANOVA and Pearson’s r test were used.

In addition, to further study how Tc1/Tc2 functioned in intrauterine inflammation, we did intracellular staining to analyze the cytokines produced by Tc1 and Tc2, including IL4, IL13, IFN-*γ* and TNF-*α*. Gating strategy and Mean Fluorescent Intensity (MFI) were exhibited in **Figures 5A, B)**. At both 6hpi and 24hpi, the expression of TNF-*α* (p<0.01, **Figure 5C**) and IFN-*γ* (p<0.001, **Figure 5D**) in placental CD8+ T cells increased. For IL13, no significant changes were found at both 6hpi and 24hpi (**Figure 5E**). There was a significant decrease of IL4 at 24hpi (p<0,01, **Figure 5F**) but no statistical significance at 6hpi (**Figure 5F**). Furthermore, TNF-*α* in all placental cells increased (p<0.05, **Figure S2**) while no changes was observed in IFN-*γ* (**Figure S3**).

## Discussion

This is the first study investigating the functional status and subsets of CD8+ T cells infiltrating into the placenta following IUI. Our results illustrated that IUI exposure may induce placental CD8+ T cell dysfunction, which may be associated with adverse perinatal outcomes. In this study, we determined that the effector and exhausted sub-population of CD8+ T cells were altered, which may aggravate the inflammatory response in fetal-maternal interface with IUI. Moreover, we found a significantly increased ratio of Type 1 to Type 2 immune response in placental CD8+ T cells and proved that following IUI, CD8+ T cells functioned primarily *via* production of Type 1 cytokines such as IFN-γ and TNF-α.

In our previous studies, we have completed research in perinatal immune and inflammatory responses and investigated extensively how the maternal immune inflammatory responses may impact neonatal outcomes (14). We also found that there was an increase in the levels of CD8+ T‐cell infiltration into the placenta in our mouse model of IUI and prevention of this increase in CD8+ T‐cell trafficking improved postnatal neurobehavior due to the remission of placental inflammation (14, 15). In addition, some studies suggested that decidual CD8+ T cells have a mixed profile of T cell dysfunction, activation, and effector function, which allows for both immune tolerance and immunity (23) and that the decidual microenvironment reduces CD8+ T effector responses to maintain tolerance to fetal antigens (23–26). However, the subpopulation of CD8+ T cells and how they functioned specifically in the pathological and physiological process of pregnancy remained unknown. Considering that CD8+ T cells can produce IFN-γ and TNF-α, we proved their effector functions to respond to LPS-induced IUI.

Here, we illustrated that effector CD8+ T cells (CD8+CD44+ CD62L-) increase trafficking to placenta and increase transition to exhausted CD8+ T cells (CD8+ Tim-3+) following IUI. CD44 and CD62L participates in various cellular functions including lymphocyte activation, recirculation and homing, and hematopoiesis (27). The expression of CD44 implies an activated status of CD8+ T cells, characterized by enhanced proliferative capacity and an increase in cytotoxicity, as well as intensive immune function and cytokine production (27). By contrast, CD62L (also known as L-selection) is a cell adhesion molecule on leukocytes such as CD8+ T cells, which is pivotal for controlling T cells homing to lymphoid tissues and infiltrating to inflammation sites (28). When a CD62L+ CD8+ T cell enters a lymph node, it can become activated by antigen presenting cells (APC), and upon activation, CD62L is cleaved and sheds from the cell surface, allowing CD8+ T cell reenter the circulation, where they can exert their regulator or effector functions (28).

A recent study reported that CD44+CD62L-CD8+ T cells are more cytolytic and cytotoxic compared to CD44+CD62L+CD8+ T cells, exhibiting decreased survival with a loss capacity for antigen-independent self-renewal (25, 29). This was also similar with our results because with the intrauterine LPS administration, CD8+ T cells acquired an increased effector phenotype, which indicated enhanced immune and inflammatory responses, as well as increasing cytotoxicity. This subset possessed a stronger proliferative capability and produced more pro-inflammatory cytokines to further promote disorders and destructions in the placenta, which may induce lasting chronic placental inflammation associated with VUE.

We also observed a time-dependent increase of Tim-3+ CD8+ T cells in the placenta following IUI. T cell immunoglobulin and mucin domain-containing protein 3 (TIM3) is a member of the TIM family of immunoregulatory proteins, which was originally identified as a receptor expressed on interferon-γ-producing CD8+ T cells (30, 31). Recent studies suggested that Tim-3 could function as an inhibitory molecule that serves to limit IFN-γ-driven inflammation (31–33) and could serve as a marker of exhausted CD8+ T cells, indicating reduced proinflammatory cytokine production and impaired cytotoxicity to control antigen or stimuli (30, 34).

Our results showed that 24 hours post LPS intrauterine administration, rather than 6 hours, Tim3+ CD8+ T cells increased in the placenta. These results indicated the abnormal function of CD8+ T cells, the limited production of immune regulators and elevated numbers of terminally differentiated IFN-γ-producing T cells. These pathological changes were probably associated with placental destruction and dysfunction related to VUE, worse neonatal outcomes including perinatal or postnatal brain injury (15). However, the impact of Tim3+CD8+ T cells on the neonates and infants directly exposed to IUI requires further study.

Some studies also found that the surface expression of Tim-3 on T cells is low in patients with autoimmune diseases compared with healthy controls. In another study, during chronic *M. tuberculosis* infection in mice, TIM3 expression on CD8+ T cells was elevated (30). Similarly, upregulated TIM3 expression was found on total CD8+ T cells in patients with tuberculosis (30, 35). Further *in vitro* experiments demonstrated that Tim-3+ T cells interact with galectin 9 on macrophages, which triggers IL-1β production by macrophages (36, 37). Our previous study showed that an elevated level of IL-1β in the placenta may exacerbate placental inflammation and induce adverse neurobehavioral outcomes in offspring (20). In accordance with this data, increasing levels of IL-1β in the placenta could also be mediated by increased placental Tim3+ CD8+ T cells indirectly in addition to activation of the TLR4 pathway.

Consequently, Tim3+CD8+ T cells may play a dual role in pathological pregnancy with IUI exposure. On one hand, increased Tim3+CD8+ T cell infiltration into the placenta may exacerbate placental inflammation by indirect interaction and communication with other cells including macrophages, resulting in worse microenvironments for the survival and development of the fetus. On the other hand, Tim3+CD8+ T cells trafficking to the placenta may aggravate toxic effects upon the placenta, since Tim3+ expression on CD8+ T cells is limited to terminally differentiated IFN-γ-producing T cells. Increased Tim3+ expression indicates an elevated frequency and enhanced IFN-γ-producing capacity of Tc1. This is also consistent with our results that Type 1 CD8+ T cells (Tc1) frequency and IFN-γ release in CD8+ T cells increased with LPS treatment after 6 hours and 24 hours, which showed aggravating toxic effects on placenta. In the future, more research needs to be designed to identify the specific mechanisms for Tim+CD8+ T cells in VUE.

We also observed an imbalance of Tc1/Tc2 and altered cytokine profiles in the placenta in mouse model of IUI. Both Tc1 and Tc2 display predominantly perforin-dependent cytolysis *in vitro*, but Tc2 exhibits reduced cytotoxic activity in comparison with Tc1 (12). Tc1 utilizes both perforin- and Fas/FasL-based killing pathways, whereas Tc2 cells primarily utilize perforin-mediated cytolysis. This means Tc1 may trigger IFN-γ primed macrophage populations to secrete pro-inflammatory cytokines such as IL-1β, which promotes aggravating intrauterine inflammation and contributes to placental dysfunction. One recent study suggested that the clearance of Brucella requires IFN-γ-mediated type-1 T cell (Th1/Tc1) immune responses (38, 39), while the IL-4, IL-10, Th2/Tc2 response determines susceptibility to Brucella and disease severity (40, 41). In another study, *in vitro*-generated tumor-reactive Tc2 cells were less efficient in eliminating lung tumors than Tc1 cells (42). IL-4 produced by CD8+ T cells inhibits IFN-γ production by CD8+ T cells and reduces cytotoxicity (43). Thus, IL-4-producing-CD8+ T cells may provide a suppressor or anti-inflammatory function in IUI associated lesions including VUE.

Taken together, Tc1 cells may have roles as effector cells and Tc2 as immunomodulatory cells. One study reported that there was increased IL-2 and IL-12 and reduced IL-4 immunostaining in VUE placentas from stillborn infants, and immune responses in the VUE lesions bias towards Th1-type response (13). It was also demonstrated that the presence of CD8 T cells in the VUE lesions may contribute to both the fetal-placental vasculopathy observed in VUE and the increased level of apoptosis in intra-villous cells (8, 13, 44, 45). In pregnancy, a bias of T cell immunity towards type 2 is thought to be critical for normal pregnancy (46–49). Pathological pregnancies, such as pre-eclampsia and VUE, may be characterized by type 1 immune dominance (13). In this paper, we first investigated the percentage and ratio of Tc1 and Tc2 in placenta in pathological pregnancies using an IUI mouse model.

An imbalance between the production of Tc1 and Tc2 cytokines contributes to an impairment in T cell-mediated immunity, which may ultimately impact anti-bacterial immune responses (41). In this study, we observed a loss of homeostasis between placental Tc1, Tc2, and their cytokine production following LPS intrauterine administration. Our results showed that compared to control dams, a higher percentage of Tc1 and higher level of cytokines IFN-*γ*/TNF-*α* produced by Tc1 were observed in LPS-treated dams. However, a lower percentage of Tc2 and IL-4 level were observed in LPS-treated dams. Our study also revealed that Tc1/Tc2 ratio increased significantly following intrauterine LPS exposure, reflecting a general skew in the immune response away from type 2 and toward type 1, indicating the activation of the aggravating immune and inflammation responses in the placenta associated with VUE.

This data is consistent with the limited regulation of the inflammatory properties of CD8+ T cells, supporting and explaining our previous studies that the depletion of CD8+ T cells would alleviate perinatal brain injury and improve postnatal behavior (15). Our findings, in conjunction with previous investigations, suggests that immunological changes in Tc1/Tc2 ratios may promote an immune-activation condition in dams with IUI exposure. An anabatic inflammatory response developed in placenta following IUI exposure, which may not be essential but has a major influence on the processes of IUI-induced fetal brain injury, placental dysfunction and destruction that are associated with VUE.

In addition, we observed differences in T cell infiltration in placenta between two timepoints. In PBS-injected animals but not in LPS-injected animals, T cells increased at E18.5 compared to E17.5, which could be supposed that T cells function as a regulator in normal parturition process while LPS administration induced a dysfunction. Furthermore, with LPS injection, CD8+ T cell and its exhaustion exhibited changes 24 hours after surgery. With LPS administration, CD8+ T cells decreased at E18.5 while exhaustion increased at E18.5 compared to E17.5, which could be speculated that LPS induced CD8+ T cells to rise earlier and also contributed to exhaustion of CD8+ T cells during parturition, amplifying inflammatory responses and worsening the fetus outcome. Tc1 showed increase during parturition regardless LPS injection, but higher frequency was observed in LPS-injected animals. The results demonstrated that Tc1 response played a vital role in normal parturition but LPS administration may amplify this inflammatory response which may contribute to fetal demise.

Despite the meaningful observations, our study had some limitations. First, we did not study how maternal Tc1 or Tc2 impact fetal brain development and placenta functions directly. Further experiments are designing to investigate how Tc1 or Tc2 alone contributes to fetus exposure to IUI. Second, we did not know if maternal CD8+ T cells infiltrated fetal organs. It needs to be investigated what subtype of CD8+ T cells are trafficked to fetal tissue and how it affects the long-term outcomes of the offspring exposure to IUI. Third, we do not know the impact of placental Tc1/Tc2 on long term sequalae for both mothers and children as well as potential mechanisms. In the future, we need to design cohort studies and perform further experiments on human placenta to determine the relationship between different type of CD8+ immune responses in the placenta and long-term effect caused by Tc1/Tc2 imbalance and CD8+ T cell dysfunction.

## Conclusion

A greater understanding of the immune and inflammatory responses associated with VUE may be beneficial to the development of a diagnosis to enable antenatal detection of VUE. Our study characterized the status and properties of placental CD8+ T cells following IUI, the main effector cells involved in VUE. In addition, we identified specific CD8+ T cell subtypes and have provided evidence for an increase of Type 1 cytokines produced by CD8+ T cells in a mouse model of IUI. This data presents a novel insight into the pathogenesis of VUE and provides solid foundations for studying basic mechanisms to determine the specific impact of CD8+ T cells on placental function.

## Data Availability Statement

The raw data supporting the conclusions of this article will be made available by the authors, without undue reservation.

## Ethics Statement

The animal study was reviewed and approved by the Animal Care and Use Committee of the Johns Hopkins University.

## Author Contributions

IB and JLi conceptualized the experiments. IB, JLi, and JLe designed the experiments. IB, AL, JLi, YL, and JLe conducted the experiments. JLi, YL, and JLe analyzed the data. JLi graphed and organized the data and figures. IB and JLi interpreted the data and wrote the manuscript. IB, JLi, and SP edited the manuscript. All authors contributed to the article and approved the submitted version.

## Funding

This work was supported by the Integrated Research Center for Fetal Medicine Fund and Sheikh Abdullah Bugshan Fund (IB).

## Conflict of Interest

The authors declare that the research was conducted in the absence of any commercial or financial relationships that could be construed as a potential conflict of interest.

## Publisher’s Note

All claims expressed in this article are solely those of the authors and do not necessarily represent those of their affiliated organizations, or those of the publisher, the editors and the reviewers. Any product that may be evaluated in this article, or claim that may be made by its manufacturer, is not guaranteed or endorsed by the publisher.

## Supplementary Material

Supplementary Figure 1 Live and aborted fetuses in each cage were measured. Offspring viability was significantly decreased in LPS-administrated dams compared to that in PBS-treated dams at 6hpi or 24hpi (****p < 0.0001; Chi-squared test; At 6hpi, LPS: 76% viable, n = 68; Control: 100% viable, n = 40. At 24hpi, LPS: 27% viable, n = 113; Control: 98% viable, n = 40).

Supplementary Figure 2 IFN-γ in placenta. There was no change for IFN-*γ* in placenta.

Supplementary Figure 3 TNF-α in placenta. At 24hpi, TNF-α in placenta was increased (**p < 0.01, n = 4 in each group).
