# Supplementary figures and images for: Type 1 Cytotoxic T Cells Increase in Placenta after Intrauterine Inflammation
Source: Front Immunol. 2021 Sep 8;12:718563. doi: 10.3389/fimmu.2021.718563 (PMC8456007; doi:10.3389/fimmu.2021.718563)

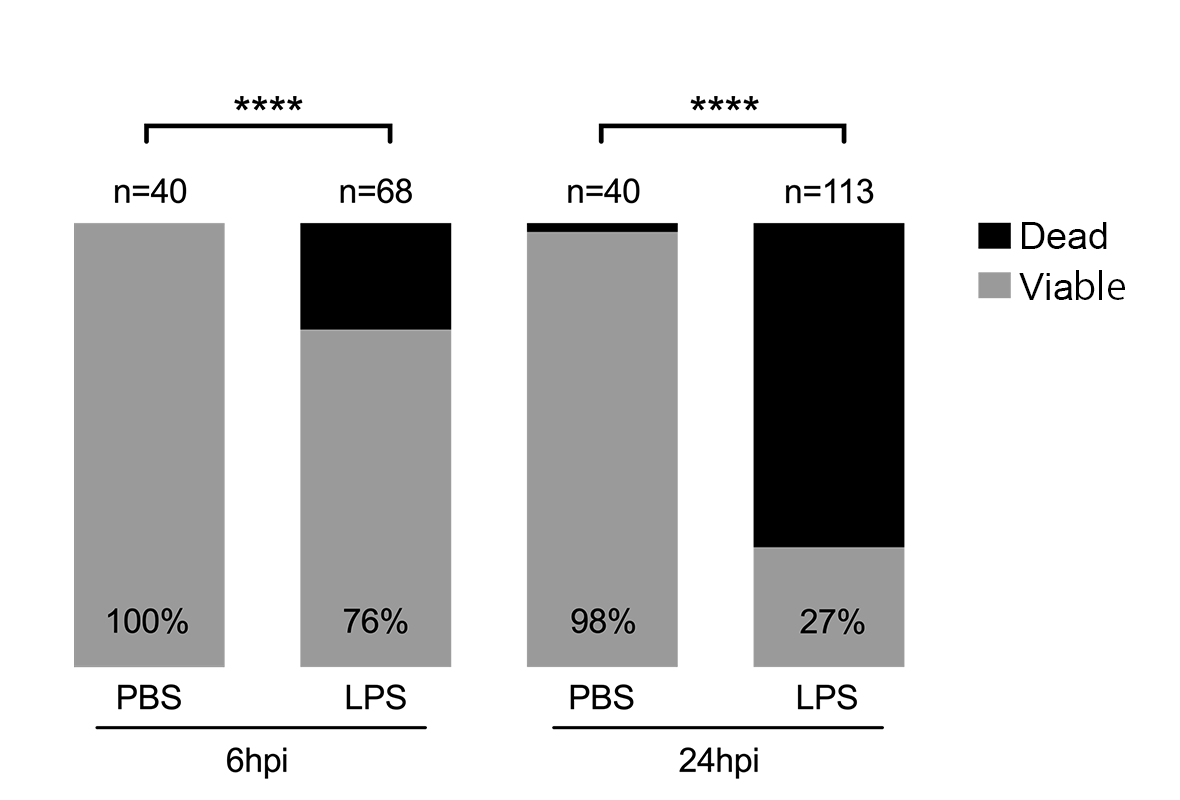

Supplement: Supplementary Figure 1 — Live and aborted fetuses in each cage were measured. Offspring viability was significantly decreased in LPS-administrated dams compared to that in PBS-treated dams at 6hpi or 24hpi (****p < 0.0001; Chi-squared test; At 6hpi, LPS: 76% viable, n = 68; Control: 100% viable, n = 40. At 24hpi, LPS: 27% viable, n = 113; Control: 98% viable, n = 40). [file Image_1.tif]

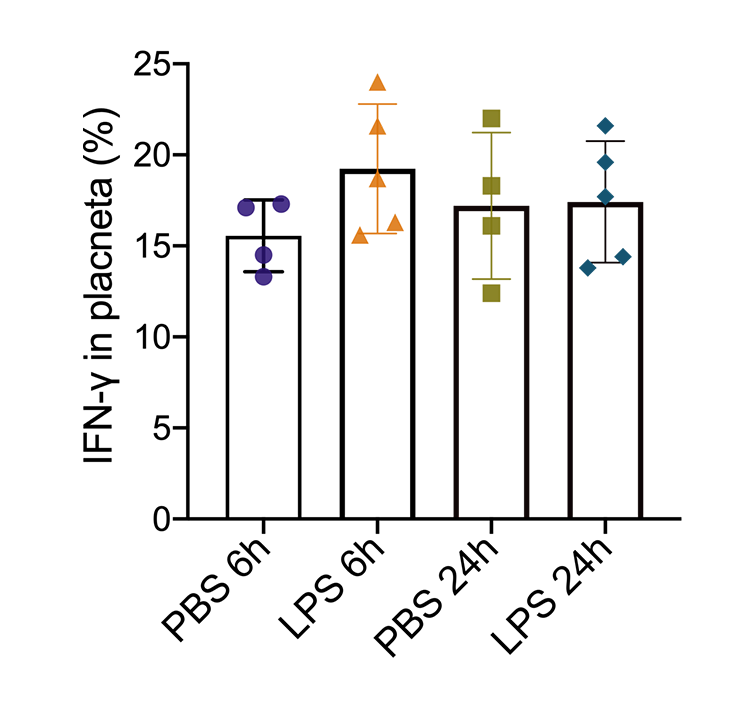

Supplement: Supplementary Figure 2 — IFN-γ in placenta. There was no change for IFN-γ in placenta. [file Image_2.tif]

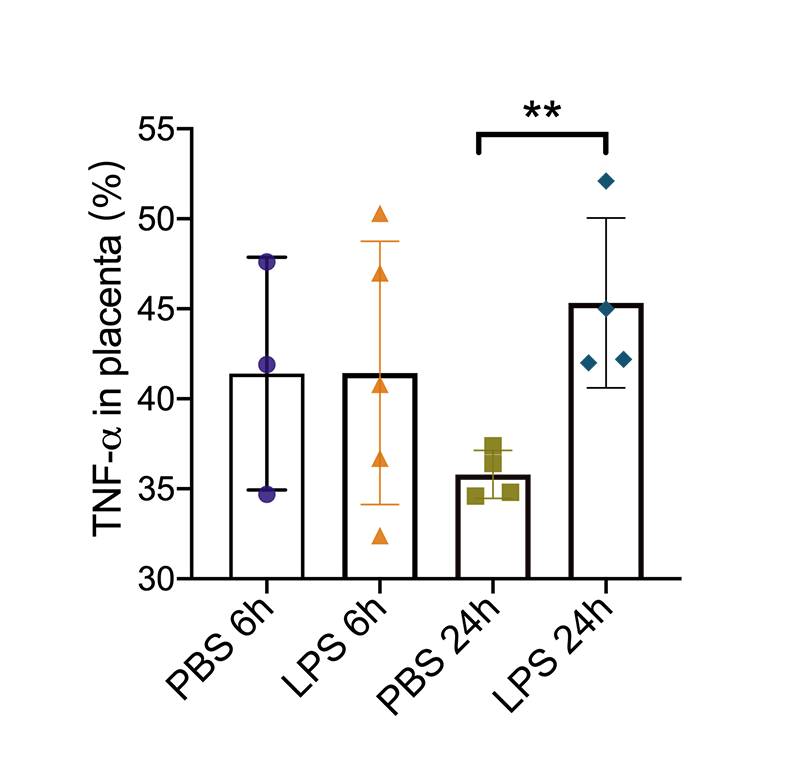

Supplement: Supplementary Figure 3 — TNF-α in placenta. At 24hpi, TNF-α in placenta was increased (**p < 0.01, n = 4 in each group). [file Image_3.tif]
